# Erdheim-Chester disease with chorioretinal and orbital involvement: a case report

**DOI:** 10.5935/0004-2749.2022-0151

**Published:** 2023-03-08

**Authors:** Kubra Serefoglu Cabuk, Adem Tellioglu, Gamze Ozturk Karabulut, Senay Asik Nacaroglu, Korhan Fazil, Tuğrul Elverdi, Muhittin Taskapili, Safak Karslioglu

**Affiliations:** 1 Ophthalmic Plastic & Reconstructive Surgery, University of Health Sciences Beyoglu Eye Training and Research Hospital, Istanbul, Turkey; 2 Ophthalmology Department, Memorial Hospital, Faculty of Medicine, Arel University; 3 Ophthalmology Department, Faculty of Medicine, Istanbul Medipol University, Istanbul, Turkey; 4 Oculoplastic and Orbital Surgery and Ocular Oncology Center, Istanbul, Turkey; 5 Division of Hematology, Department of Internal Medicine, Cerrahpasa Faculty of Medicine, Istanbul University-Cerrahpasa, Istanbul, Turkey

**Keywords:** Hemic and lymphatic diseases, Histiocytosis, Histiocytosis, non-Langerhans cell, Erdheim-Chester disease, Retinal diseases, Orbital diseases, Humans, Case reports, Doenças sanguíneas e linfáticas, Histiocitose, Histiocitose de células de Langerhans, Doença de Erdheim-Chester, Doenças retinianas, Doenças orbitárias, Humanos, Relatos de casos

## Abstract

A 42-year-old woman presented with bilateral proptosis, chemosis, leg pain, and vision loss. Orbital, chorioretinal, and multi-organ involvement of Erdheim-Chester disease, a rare non-Langerhans histiocytosis, with a negative BRAF mutation was diagnosed based on clinical, radiological, and pathological findings. Interferon-alpha-2a (IFNα-2a) was started, and her clinical condition improved. However, 4 months later, she had vision loss with a history of IFNα-2a cessation. The same therapy was administered, and her clinical condition improved. The Erdheim-Chester disease is a rare chronic histiocytic proliferative disease that requires a multidisciplinary approach and can be fatal if left untreated because of multisystemic involvements.

## Abstract (pt)

Uma mulher de 42 anos apresentou proptose bi-lateral, quemose, dor nas pernas e perda de visão. Com base em achados clínicos, radiológicos e patológicos, foi diag-nosticada doença de Erdheim-Chester com acometimento orbitário, coriorretiniano e multiorgânico. Trata-se de uma rara histiocitose não Langerhans negativa para a mutação BRAF. Foi iniciado tratamento com interferon alfa-2a (IFNα-2a) e o quadro clínico melhorou. No entanto, quatro meses depois, a paciente apresentou perda visual após a cessação do IFNα-2a. A mesma terapia foi administrada novamente e sua condição clínica melhorou novamente. A doença de Erdheim-Chester é uma doença proliferativa histiocítica crônica rara que necessita de uma abordagem multidisciplinar e pode ser fatal se não tratada, devido a envolvimentos multissistêmicos.

## INTRODUCTION

We report bilateral orbital, chorioretinal, and multi-organ involvement of an unusual Erdheim-Chester disease (ECD), a rare non-Langerhans histiocytosis, oncogenic mutations in the mitogen-activated protein kinase pathway found in most of the cases (MAP2K1, ARAF, NRAS, KRAS, BRAF, ALK, and NTRK1) leading to uncontrolled histiocyte infiltration and end-organ dysfunction^(1)^.

This study adhered to the tenets of the Declaration of Helsinki, and informed consent was obtained.

## CASE REPORT

A 42-year-old Moldovian female patient presented to the University of Health Sciences Beyoglu Eye Hospital because of bilateral proptosis, leg pain, and vision disturbances in May 2019. The patient’s medical history was significant for a newly diagnosed diabetes insipidus and auto-immune thyroiditis with normal thyroid functions.

**Figure 1:**
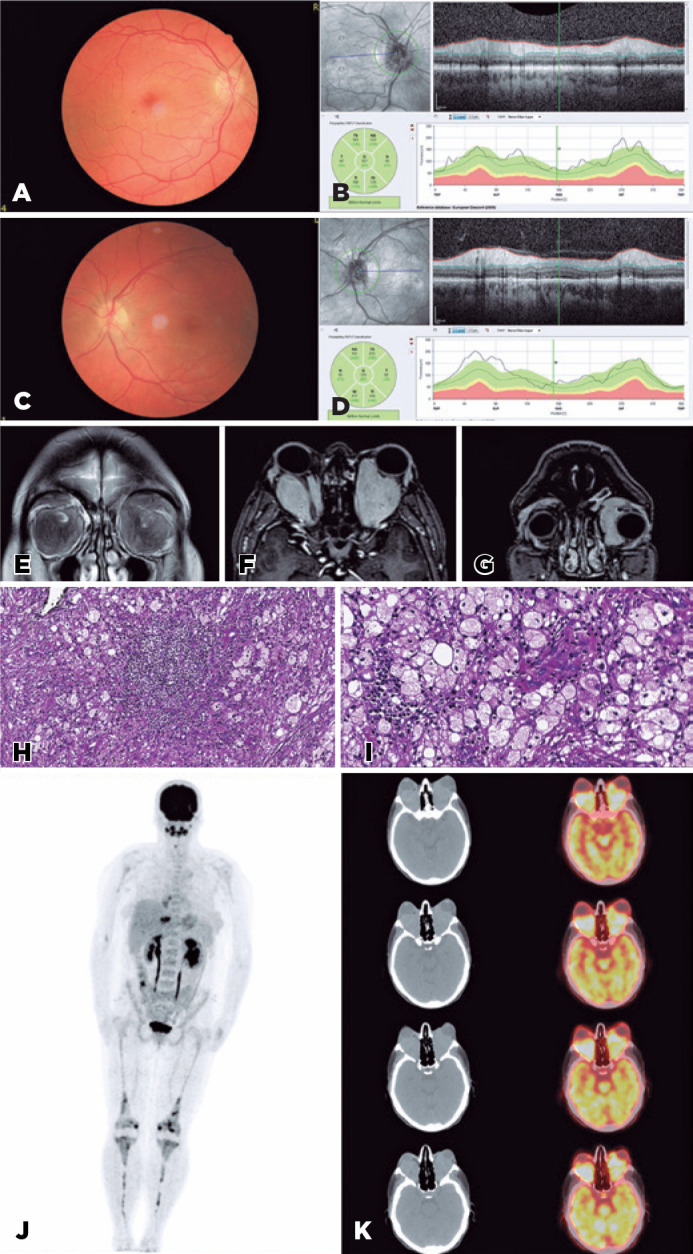
(A, C) Color fundus photographs: Bilateral blurring of the optic disc margins and mild paling at the left temporal optic disc. (B, D) Macular RPE changes and choroidal folds are more prominent in infrared images. OCT shows edematous peripapillary nerve fiber layer and subretinal fluid at the nasal segment of the left optic nerve. (E, F, G) T1-weighted precontrast axial (E) and coronal (F) sections show low-signal intensity homogeneous lesions that fill up the intraconal space and spill over the extraconal space. Postcontrast T1-weighted fat-suppressed axial (G) sections demonstrated splaying of the extraocular muscles, flattening of the posterior right globe, and lateral displacement of the left globe by the infiltration exceeding the equator and/extending to the insertion of left medial rectus (H, I) Hematoxylin and eosin staining demonstrates abundant infiltration of foamy histiocytes within a background of fibrosis and inflammation (×100, ×400). (J) 18-FDG PET/CT shows bilateral skeletal FDG uptake especially in femur and tibia (K) Hypermetobolic bilateral retroorbital soft tissue mass with intense FDG uptake.

The initial examination revealed conjunctival hyperemia and bilateral chemosis with proptosis (28/29 mm Hertel, base: 110 mm). Relative afferent pupil defect was positive in the left eye. Ishihara’s color plate reading was 4/16 in the right eye (OD) and 2/16 in the left eye (OS). Ocular movements were free in all directions bilaterally. Visual acuity (VA) values were 20/40 OD and 20/50 OS. Results of the biomicroscopic anterior segment evaluation were normal, but the blurring of the optic disc margins and macular pigment epithelial changes were evident bilaterally and choroidal folds at OD, as confirmed by optical coherence tomography (OCT) and fundus photo (Figures 1A-1D). The patient underwent orbital magnetic resonance imaging (MRI) with contrast (Figures 1E-1G).

Laboratory investigations including serum glucose, urea, creatinine, alanine aminotransferase, aspartate aminotransferase, and hemogram were unremarkable. In addition, 1 g/day methylprednisolone was given to the patient intravenously (IV) for 3 days for optic neuropathy till biopsy. Transseptal orbital incisional biopsy was performed via the left medial lid crease incision (Author SK). Pathological specimens are presented in Figures 1E-1I.

Clinical, imaging, and pathological findings were consistent with ECD. The patient consulted with the hematology department (Author TE). Results of the laboratory investigations are summarized in Table 1. Bone marrow biopsy was performed, and BRAF mutation found negative.

**Table 1:** Signs and symptoms and associated imaging studies

Findings	Signs and symptoms	Imaging studies - Literature	Imaging studies - our patient
Skeletal (90%)	Bone pain especially in the lower limbs	99mTc-bone scintigraphy: symmetrical uptake in the lower limbs	18F-FDG PET/CT: osteosclerotic changes and intense FDG uptake in the skeletal system, especially in the long bones
Neurological (50%)	Diabetes insipidus	Brain MRI with contrast: Gadolinium-enhancing lesions; involvement of the dentate nuclei of the cerebellum and pons Fundus examination revealing papilledema or retinal nerve fiber layer thickness	MRI of the hypophysis: normal 18F-FDG PET/CT: Hypermetobolic focus at the left anteromedial cerebellum
	Panhypopituitarism		
	Papilledema		
Cardiovascular (45%)	Pericardial pain Cardiac tamponade Cardiac failure Myocardial infarction	Cardiac MRI and cine-MRI: pseudotumoral lesion of the right atrium or right atrioventricular sulcus; pericoronarial infiltration; circumferential infiltration of the thoracic and abdominal aorta (“coated aorta”)	Echocardiography: hyperecogenic thickening of the pericardium. Effusion in the pericardium 3 mm in the widest area
Renal (30%)	Dysuria Abdominal pain	Abdomen CT scan/MRI: retroperitoneal and perirenal space infiltration (“hairy kidney”); hydroureteronephrosis Angio-CT or Doppler-US	18 FDG PET/CT: Thickening at the cortex and periphery of the left kidney
Orbital (25%)	Exophthalmos Visual impairment	Orbital MRI with contrast: intraorbital T2-hypointense enhanced pathological tissue	Orbital MRI with contrast: T1 bilateral low-signal intensity, homogenous lesion filling the retroorbital space. Postcontrast T1; splaying of the extraocular muscles, lateral displacement of the left globe by the infiltration pre-equator. T2-hypointense enhanced pathological tissue

The patient was started on interferon-alpha-2a (IFNα-2a) at a dose of 3 MIU 3 times per week. On week 3, the patient’s vision was 20/20, Ishihara readings were 16/16 bilaterally, and Hertel readings were 24/25 mm. The patient returned to Moldova under IFNα-2a therapy.

**Figure 2:**
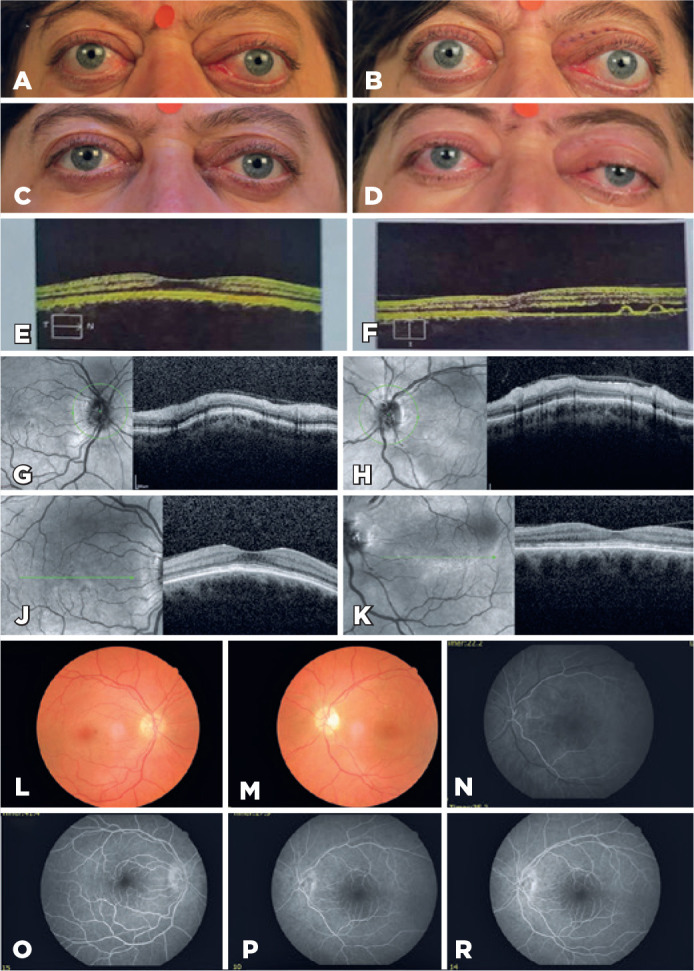
(A) Clinical photograph at the presentation. (B) After pulse steroid, before interferon-alpha therapy (C), 3rd week of interferon-alpha 2a. (D) After the cessation of interferon treatment (approximately after 7 weeks of initialization). The photograph showed proptosis and cushingoid appearance. (E, F) M-OCT before injection shows no pathology in OD and subretinal fluid and pigment epithelial detachment (PED) in OS. (G-K) M-OCT and G-OCT show RPE irregularities in both eyes, but there is no subretinal fluid or PED after periocular steroid injection. (L, M) Color fundus photographs show paling of the temporal of the left optic disc, yellow-orange macular mottling greater in OS. (N-R) Fundus fluorescein angiography shows delayed choroidal and retinal filling, two little focal leakages at the superior-nasal peripapillary in OS, and macular flowerlike hyperfluorescence in OD.

Four months later, the patient presented with vision loss in the left eye. On examination, the patient’s vision was 20/25 in OD and no light perception in OS. Hertel readings were 26/28 mm. Fundus examination revealed paling of the temporal left optic disc and yellow-orange macular mottling greater in OS. The patient had a cushingoid appearance, depressed behaviors, and social withdrawal. The patient reported temporary improvement of vision, weakness, inability to walk, and a fall resulting in hospitalization. The patient’s relatives reported a history of cessation of IFN treatment (approximately after 7 weeks of initialization), a decrease in vision 3 weeks later, followed by a periocular steroid injection by the ophthalmologist in the patient’s country. OCT recordings and fluorescein angiography before injection are shown in Figures 2E-2F and 2N-R.

IFNα-2a 3 MIU 3 days/week sc was started again with 1 mg/kg oral prednisolone. Proptosis regressed to 24/26 mm, OD vision improved to 20/20, but the OS vision did not improve on the 10th day of IFNα-2a.

Although the need for strict multi-system monitoring and neurologic and psychiatric consultations were emphasized, the patient ceased coming for follow-up examinations and returned to her country.

## DISCUSSION

ECD is a rare non-Langerhans cell histiocytosis that causes multisystemic involvement with end-organ infiltration. Early diagnosis and treatment are very important to lessen morbidity and mortality.

There are less than 50 reports of orbital involvement in the literature and favor a poor prognosis as in central nervous system (CNS) involvement^(2)^. Patients often present with painless bilateral progressive proptosis, sometimes accompanied by decreased vision and diplopia, as in our case. Chemosis, proptosis, ophthalmoplegia, retinal striae, and papilledema may be seen on examination, as in our case^(3,4)^.

Intraocular involvement is also rarer than orbital involvement. Choroidal infiltration and serous retinal detachment may be seen, as evident in our case^(5-7)^. To our knowledge, we present the first case of ECD with orbital and chorioretinal involvement responding to IFN treatment. Contrast-enhanced orbital MRI showed infiltrating of not only the anterior orbit but also the entire orbit, unlike other histiocytes^(8)^.

On presentation to the ophthalmology department, the patient had pain in the legs suspecting bone infiltration, which was confirmed by positron emission tomography/computed tomography (PET/CT) as osteosclerotic changes and intense fluorodeoxyglucose (FDG) uptake in the skeletal system, especially in the long bones. Although diabetes insipidus indicated pituitary infiltration, MRI results were normal. CNS involvement is seen in 51-92 patients^(4)^. Cranial MRI showed hypermetobolic focus at the left anteromedial cerebellum. This lesion might cause ataxia and a fall in Moldova. Echocardiography was performed because of frequent cardiac involvement and mortality risk.

BRAF mutation is important in the treatment because successful results were reported with targeted BRAF inhibitor therapy, vemurafenib, in many patients with orbital ECD^(9,10)^. Unfortunately, the patient was BRAF (-). According to consensus guidelines for BRAF-negative cases, the first-class therapy has been reported as pegylated IFN (PEG-IFN) alfa and anakinra. Our patient had a visibly good response to IFN within 1 month; however, after she returned to her country, maybe because of depression and disinhibition caused by the disease and/or IV steroids, the patient discontinued the treatment, and the patient’s doctors continued the treatment with subtenon and steroids administered IV. The cessation of IFN possibly caused the relapse.

The development of pigment epithelial detachments (PED) and central serous retinopathy (CSR) immediately after discontinuation of IFN therapy suggests that INF influences PED and CSR in ectatic corneal disease. Significant reduction in proptosis with the reintroduction of IFN suggests continued good clinical response, although vision did not recover in OS.

The diagnosis and treatment of these patients have many contributions to medicine and science beyond individual healing. Therefore, financial facilitating conditions should be considered in the diagnosis and treatment of rare diseases.

ECD is a rare chronic histiocytic proliferation disease with a poor prognosis and delayed diagnosis. A multidisciplinary approach, mainly ophthalmology and hematology, is necessary for its management.
